# Biological Activity of Cyclic Peptide Extracted from *Sphaeranthus amaranthoides* Using De Novo Sequencing Strategy by Mass Spectrometry for Cancer

**DOI:** 10.3390/biology12030412

**Published:** 2023-03-07

**Authors:** Swarnalatha Yanamadala, Sivakumar Shanthirappan, Sidhika Kannan, Narendran Chiterasu, Kumaran Subramanian, Lamya Ahmed Al-Keridis, Tarun Kumar Upadhyay, Nawaf Alshammari, Mohd Saeed, Guru Prasad Srinivasan, Rohini Karunakaran

**Affiliations:** 1 Department of Biotechnology, Sathyabama Institute of Science and Technology, Chennai 600119, India; 2 Department of Gunapadam, National Institute of Siddha, Chennai 600047, India; 3 Centre for Nanoscience and Nanotechnology, Sathyabama Institute of Science and Technology, Chennai 600119, India; 4 Centre for Drug Discovery and Development, Sathyabama Institute of Science and Technology, Chennai 600119, India; 5 Department Biology, College of Science, Princess Nourah Bint Abdulrahman University, Riyadh P.O. Box 84428, Saudi Arabia; 6 Department of Biotechnology, Parul Institute of Applied Sciences and Centre of Research for Development, Parul University, Vadodara 391760, India; 7 Department of Biology, College of Science University of Hail, Hail P.O. Box 2440, Saudi Arabia; 8 Centre for Research, Bharath Institute for Higher Education and Research, Chennai 600073, India; 9 Unit of Biochemistry, Faculty of Medicine, AIMST University, Bedong 08100, Malaysia; 10 Centre for Excellence for Biomaterials Science, AIMST University, Bedong 08100, Malaysia; 11 Department of Computational Biology, Saveetha School of Engineering, SIMATS, Chennai 602105, India

**Keywords:** AMPs, cyclic peptide, orbitides, de novo sequencing, zebrafish, cytotoxicity, anti-proliferative

## Abstract

**Simple Summary:**

Cancer therapy today has benefited from multifaceted approaches in early detection and diagnosis, but weak prognosis still hinders progress, as it is a barricade for guaranteed successful treatment. The present study checks the anticancer properties of AMPs, or antimicrobial peptides, isolated from *Sphaeranthus amaranthoides*, a traditional medicinal plant in a fibroblast cell line. A technique named ‘de novo’ sequencing was used for identifying the biological potential of the isolated molecule. That molecule was administered in zebrafish embryos. The zebrafish was used as a model organism, as it has close proximity with humans. One specific peptide biomolecule out of 86 peptides showed promising anticancer properties. Therefore, from the results, that specific biomolecule, upon detailed analyses of other parameters, can be taken for upscaling purposes in the pharmaceutical sector for early cancer therapy.

**Abstract:**

Though there are several advancements and developments in cancer therapy, the treatment remains challenging. In recent years, the antimicrobial peptides (AMPs) from traditional herbs are focused for identifying and developing potential anticancer molecules. In this study, AMPs are identified from *Sphaeranthus amaranthoides*, a natural medicinal herb widely used as a crucial immune stimulant in Indian medicine. A total of 86 peptide traces were identified using liquid-chromatography–electrospray-ionisation mass spectrometry (LC-ESI-MS). Among them, three peptides were sequenced using the manual de novo sequencing technique. The *in-silico* prediction revealed that SA923 is a cyclic peptide with C-N terminal interaction of the carbon atom of ASP7 with the nitrogen atom of GLU1 (1ELVFYRD7). Thus, SA923 is presented under the orbitides class of peptides, which lack the disulfide bonds for cyclization. In addition, SA923, steered with the physicochemical properties and support vector machine (SVM) algorithm mentioned for the segment, has the highest in silico anticancer potential. Further, the in vitro cytotoxicity assay revealed the peptide has anti-proliferative activity, and toxicity studies were demonstrated in *Danio rerio* (zebrafish) embryos.

## 1. Introduction

Cancer is one of the deadliest diseases and generates a high mortality rate compared to numerous other diseases. It causes about 6 million deaths per year. Cancers are characterized by uncontrolled cell growth, local tissue invasion, and distant metastases. Cancer is caused by internal factors (tobacco, chemicals, radiation, and infectious organisms) and external factors (mutations, hormones, and immune disorders). More than 60% of the anticancer drugs currently used are derived from natural sources such as plants, microorganisms, and marine organisms. Molecules derived from natural sources have played a vital role in the invention of lead compounds for the development of conventional drugs to treat a range of human diseases [1].

Plants are composed of a rich source of biologically active substances that can be involved in various applications [2]. Among all the substances produced by plant origin, antimicrobial peptides (AMPs) are of crucial interest because the AMPs serve as a defense barrier, killing pathogens by interaction with phospholipids and membrane permeabilization [3]. Plants have multi-level immune systems to combat stress, drought, pathogens, and pests. As the primary line of protection, plants produce AMPs as the constituent part of the innate immune system [4]. Among the diverse defense mechanisms in plants, chemical defense plays an important role. Once the invader or phytopathogen recognises the activator of the plant organs, an enormous arsenal of defensive compounds is produced [5]. The production of anti-herbivore compounds, enzymes, and AMPs thwart the colonization of the phytopathogens and reduce the damage of plant tissues. In the midst of the compounds produced during defense, AMPs are of prime importance.

AMPs are small peptides ranging between 2–10 kDa in size [6]. Most of the AMPs have similar properties (cationic and amphipathic); however, they have discrete structures, functions, and modes of action. Peptide therapeutics were increasingly high from 1980 to 2010. Despite low stability and poor oral bioavailability, perhaps less attention was specified for peptide research from 2010 to 2015 [7]. In recent years, peptide research has been booming due to the advancement of the peptide delivery mechanism using liposome technology, nano-formulation, and coatings with biopolymers. Alternative strategies, such as peptide engineering, amino acid replacement/substitution, and peptide conjugation, enable us to overcome a few limitations, such as solubility, hydrophobicity, and length.

To date, 63 peptides have been approved as drugs, and the peptide therapeutic market value has reached USD 23 billion in the year 2020 [8]. Thus, peptide identification from different species, such plants, animals, and fishes, are of great interest due to their distinctive characteristic and natural occurrence upon selective pressure. However, plant-based AMPs are notably appealing, owing to the compact spatial structure attained by the intramolecular disulfide bonds. Based on these properties, plant-based AMPs are of great interest because they are identified with good biological activity [9]. However, wild plants are signified as valuable and consequently are a poorly explored source for AMP identification.

The genus *Sphaeranthus* sp. (*Asteracea*) is a group of herbal plants, in which 33 species are distributed worldwide and 3 species are present in India. *Sphaeranthus indicus*, *Sphaeranthus africanus*, and *Sphaeranthus amaranthoides* are geographically located in India. The genus is well-known for its ethnomedical properties. Among the species, *S. indicus* has been investigated vastly for different properties, such as anti-inflammatory, antimicrobial, asthma, hepatoprotective, and bronchitis [10]. However, *S. amaranthoides* is reported to be more effective than *S. indicus*. In recent years, several investigations have been carried out with different parts of the *S. amaranthoides*. Previously, the flowers were used as a stimulant and for treatment of acne and dermatitis. The seed portions were used for deworming, for treating stomach ailments, and to boost appetite. Moreover, *S. amaranthoides* has been used as an antimicrobial, hepatoprotective, rejuvenation, anti-inflammatory, and anticancer agent [11].

The whole herb is used as a source for traditional medicine. Owing to its immense medical application, each part of this plant has been discretely investigated to reveal its bioactive potential. The plant is under the least-concern category in the IUCN (International Union for Conservation of Nature). It is an organization dedicated to the preservation of nature and natural resources. The purpose of the IUCN is to “influence, promote, and help societies across the world in conserving nature”. It also ensures that any use of natural resources is ecologically sustainable. Due to the enormous biological applications, the herb is in great demand, which can lead to the depletion of the primary habitats. Therefore, several attempts were made for in vitro micropropagation and in vitro culturing, which allow for large-scale multiplication and subsequent exploitation of *S. amaranthoides*. In accordance with micropropagation, this herb is also economically suitable to be cultivated. Phytochemical analysis has revealed that crude extract of *S. amaranthoides* is enriched with several constituents, such as alkaloids, steroids, flavonoids, and tannins [12]. Additionally, ethyl acetate extract and the essential oil of *S. amaranthoides* has shown a toxic effect against the dengue mosquito vector *Aedes aegypti* [13]. The chloroform extracts of *S. amaranthoides* have shown cytotoxicity and anti-tumour effects. In addition, chrysosplenol D, a flavonoid, was identified from *S. amaranthoides* with a chemoprotective effect [14]. However, there are no details regarding the AMPs from *S. amaranthoides*. Hence, the present study is focused on the identification of peptides using the de novo sequencing technique. Further, in silico studies were performed to reveal the physicochemical characteristics and structure of the peptide. In vitro anti-proliferative tests and in vivo toxicity tests were conducted to study the potency of the peptides to be used for clinical or agricultural applications.

## 2. Materials and Methods

### 2.1. Biological Materials and Extraction

The *S. amaranthoides* (as a whole herb), which is readily available as a coarse powder, was procured from the local vendors of a Siddha herbal market. The commercially available powder had a good fragrance and was brown in colour. For extraction of the peptides, the dried powder was subsequently dissolved in a 50:50 ratio of acetonitrile: MilliQ and kept at 4 °C for 48 h. The mixture was filtered and concentrated using a vacuum evaporator (RVC 2-18 CDplus).

### 2.2. LC-ESI Mass Spectrometry for the Herbal Extract

The ESI mass spectra were recorded on a Bruker Daltonics Esquire 3000 Plus Ion-Trap Mass Spectrometer attached to an Agilent 1100 Series HPLC (high-performance liquid chromatography) system. The samples were infused into the mass spectrometer either by direct injection or through an HPLC column (Agilent, Santa Clara, CA, USA, ZORBAX analytical C18 column, 150 × 4.6 mm, 5 μm, 90 Å pore size) and eluted using a binary gradient of water (0.1% TFA): acetonitrile (0.1% TFA) at a flow rate of 0.2 mL/min. Data were acquired over the *m*/*z* range of 100–2000 in the positive ion mode. To identify the number of peptide components from the *S. amaranthoides* extract, LC-ESI-MS was performed. The extract was dissolved into its respective solvents and filtered through a 0.2 µm filter. This filtrate of *S. amaranthoides* extract was maintained as stock solution to perform the mass spectrometric analysis. An aliquot of the crude extract was separated using an HPLC in a reverse-phase C18 column (Agilent ZORBAX, Santa Clara, CA, USA), and the eluent was directly infused to the coupled mass spectrometer to identify the total ions (molecules) found in the crude extract. CID fragmentation was performed to find the fragmented daughter ions. All the daughter ions obtained were examined for the respective amino acid sequence [15]. All spectral data were annotated through the mass-spectrometry software Data Analysis 4.1 (Bruker Daltonics, Bremen, Germany).

### 2.3. In Silico Characterization of the Peptides

The putative peptide derived from the *S. amaranthoides* extract was screened for its physiochemical parameters, and stability was calculated using the ProtParam tool available with Expasy. In silico methods were used in the forecasting and scheming of the anticancer peptides. Anticancer peptides often originate from antimicrobial peptides. They are cationic in nature and are considered safe to normal cells but are toxic to bacteria. The major determinant in the annihilation of cancer cells is considered to be the electrostatic interactions of cationic amino acids in anticancer peptides. The high cell surface area of the cancer cells also leads to the increase in disintegration of anticancer peptides. The disorganization of the mitochondrial membrane when transferred to the cancer cells leads to programmed cell death. Moreover, the aliphatic index, protein binding interaction potential, and hydrophobicity of the peptides were estimated using the tool in the APD3 (an antimicrobial peptide database) [16]. A Basic Local Alignment Search Tool (BLAST) for protein (BLASTP) was performed against the protein sequence of the herbaceous (temperate herbaceous clade (taxid: 2233839)) database to predict the class of the novel peptides. The functional role of the peptides was determined through the AntiCP web server. The AntiCP web server was expanded in order to anticipate anticancer peptides that are highly beneficial and constructive. This server was developed based on the supportive vector machine models. The amino acid composition plays an important role in the AntiCP web server. It is a user-friendly web server [16]. Furthermore, the 3D structure of the peptides was obtained using PEPstrMOD. The predicted structure was verified using the ProSA web server for its quality [17]. The studies were carried out for the SA626, SA923, and SA905 peptides out of the 86 peptides.

### 2.4. In Vitro Cytotoxicity Assay

For determining the cytotoxic effect of the peptides, a 3-[4,5-dimethylthiazole-2-yl]-2,5-diphenyltetrazolium bromide (MTT dye) based assay was performed. The assay was performed using 3T3 cell lines. The 3T3 cell lines were considered because they could evidently grow indefinitely while being unable to initiate tumour growth. To the 96-well plates containing 100 µL media, 5 × 10^3^ cells were added, and the plates were kept at 37 °C in a CO_2_ incubator for 24 h. After the attachment of the cells, the media was aspirated and replaced with 200 µL of fresh media supplemented with different concentrations (10 ng, 20 ng, 40 ng, 80 ng, and 160 ng/ml) of the peptides. Subsequently, the plates were incubated for 24 h at 37 °C. Following the drug exposure, the cells at 12 h were incubated with 5 mg/ml of MTT at 37 °C for 3 h. Finally, the medium was removed, and the insoluble formazan product was dissolved in dimethyl sulfoxide (200 µL) and kept in a dark condition for 15 min. The insoluble formazan was quantified by measuring the absorbance at 570 nm using a multi-mode microplate reader (EnSpire, Perkin Elmer, Waltham, MA, USA). The assay was performed in triplicates.

### 2.5. Zebrafish Embryo Toxicity Test

For studying the toxic effect of the peptides, zebrafish embryos were used. Adult and healthy zebrafish were obtained from the standalone system (Aquaneering, San Diego, CA, USA). To yield the embryos, male and female zebrafish were kept in the breeding tank at 25–28 °C, with a 14–10 h light/dark-cycle photoperiod. Later, the healthy zebrafish embryos, without any visible physical defects, at 6 hpf (hours postfertilization) were used for the assay. The E3 medium was prepared by the composition of 1 × E3 embryo medium, diluted with 16.5 mL 60× stock in 1 L ddiH20. Then 100 µl of 1% methylene blue was added. Then ten embryos were poured in each well of the 24-well microtitre plate. The test wells were supplemented with different concentrations (10 ng/mL, 20 ng/mL, 40 ng/mL, 80 ng/mL, and 160 ng/mL) of the peptides, and the toxicity was assessed. The mortality and developmental deformities of the zebrafish larvae were recorded at 24, 48, and 72 hpf [15,18].

## 3. Results and Discussion

Wild plants are seldom valuable for bioactives [19,20] and are still poorly explored as sources of antimicrobials and anticancer agents. *S. amaranthoides* is a weed that grows along with paddy plants and has been explored as an important immunostimulant in the Indian medicine system. Only a few studies have been performed with *S. amaranthoides* [9]. Till now, different extracts of *S. amaranthoides* were explored for antitumor, antimicrobial, and cytotoxicity [19,21] effects. Hence, the present study aims to identify peptides from this species and explore their biological effect.

### 3.1. LC-ESI Mass Spectrometry for the Herbal Extract

**Figure 1:**
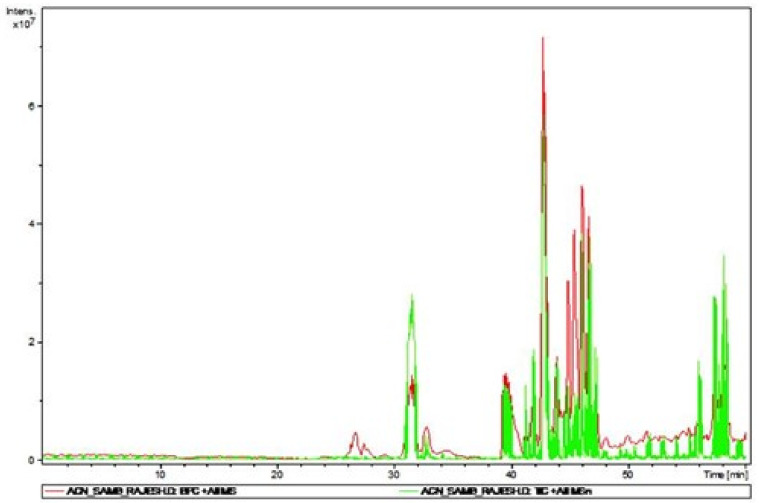
Total ion chromatogram of natural extract of *S. amaranthoides*.

The total ion chromatogram unveils the series of peptide components (Figure 1) from *S. amaranthoides*.

The elution of most of the peptides ranged from 25 min to 50 min, which indicates the acidic and neutral nature of the peptide components. Few sugar-based components were also traced from the 25–32 min elution. LC-MS investigation of the herbal extract revealed the presence of peptides in the masses, ranging from 620 to 920 Da. A total of 86 *m*/*z* traces were identified (Table 1) by analysing the LC-MS spectrum of the HPLC fraction.

By manual annotation using the de novo sequencing strategy, three novel peptides were identified from the extract. Among them, two peptides were linear (SA626 and SA905), and one was a cyclic peptide (SA923) (Table 2).

**Figure 2:**
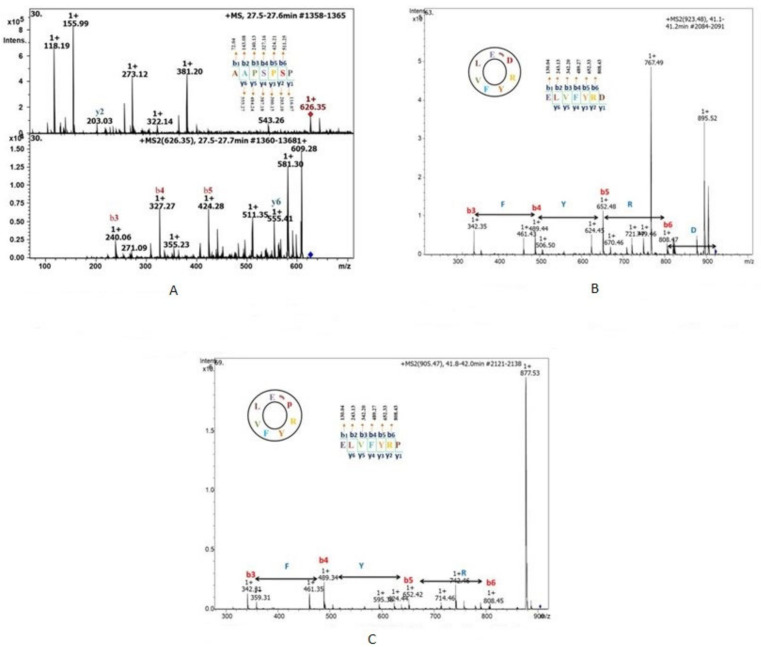
Denovo sequencing of (**A**) linear peptide SA626 from the daughter ions derived from fragmentation of singly charged (M+H) 626.35 and (**B**) cyclic peptides SA 923.4 and (**C**) SA 905.4 from the daughter ions derived from fragmentation of singly charged (M+H) 923.4 and 905 respectively.

The Mass spectrometry (MS) fragmentation data of the singly charged ion with 626.35 *m*/*z* [M+H] is presented in Figure 2A. The series of ‘b’ and ‘y’ ions for the SA626 sequence was carefully analysed, which resulted in the sequence of AAPSPSP-NH_2_. The MS2 fragmentation data of the singly charged ion with 923.48 *m*/*z* [M+H] is presented in Figure 2B. The sequence of ‘b’ and ‘y’ ions for peptide SA923 was derived unambiguously as ELVFYRD. The fragmentation data of the doubly charged ion with 905.47 *m*/*z* [M+H] is presented in Figure 2C. 

Based on the daughter ions generated, the peptide SA905’s sequence of amino acid residues was derived as ELVFYRP. The N-terminal glutamic acid interacts with C-terminal proline to form the cyclic peptide. The only difference between SA923 and SA905 is the amino acid mutation in the 7th residue. SA905 has a proline residue instead of an aspartic acid residue, which was observed with SA923. The mass spectrum also revealed different sugar-based molecules (SA1029.3, SA1013.4, SA887, SA757.2, and SA741.2), such as multiple hexose and fucose molecules.

### 3.2. In Silico Characterization of the Peptides

Computational analysis can provide insightful knowledge of the peptides, such as their amino acid composition, structure, physicochemical properties, and other functional analyses [22,23]. Based on the in-silico studies, the best candidate with potent biological activity can be further evaluated using experimental investigation and can successfully enter the drug-discovery pipeline (Table 3).

The biological activity of the peptides greatly relies on their amino acid composition, structure, and physicochemical properties. For a peptide to be considered to have antimicrobial and anticancer properties, it should encompass a hydrophobicity of 40–60% and isoelectric point of up to 10. The protein stability is an important factor for drug discovery, for which an instability index smaller than 40 is predicted as stable and a value above 40 predicts that the peptide may be unstable. Thus, among the peptides, SA923 (30.99) is predicted to be stable compared to the other peptides and suitable for pharmaceutical applications. The aliphatic index is another important feature to determine the thermostability of the protein. This index is predicted based on the relative volume of the aliphatic side chains (alanine, valine, isoleucine, and leucine) present. The GRAVY index score is the measure of the average hydrophobicity and hydrophilicity of proteins, calculated using the Kyte-Doolittle and Hopp–Woods formulas, respectively. The hydrophobicity score has an arbitrary unit, where a score below zero reveals the peptide is more likely to be derived from globular hydrophilic protein, while a score above zero reveals it is more likely a membranous hydrophobic peptide. Thus, in the present study, the peptides with scores below zero are determined to be under the class of hydrophilic peptides. The Boman index represents the protein binding potential, where a score above 2.4 kcal/mol determines the protein with the highest interaction. The peptide SA923 is predicted to satisfy this criterion with a score of 2.66 kcal/mol.

**Figure 3:**
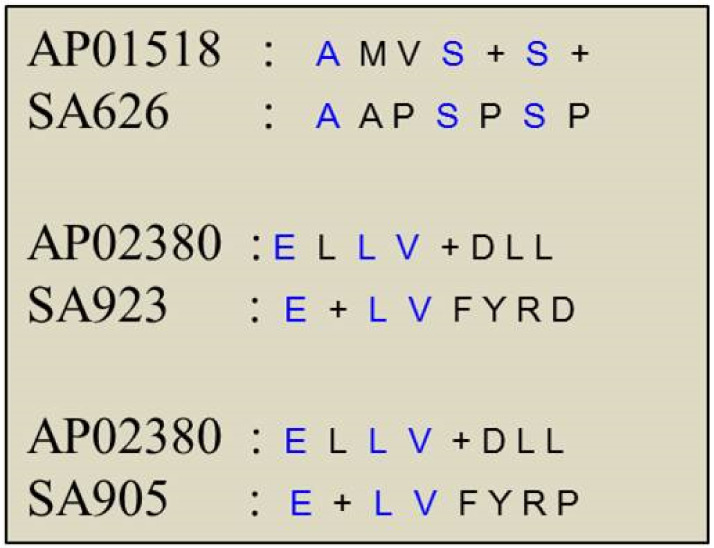
Sequence alignment of peptides SA626, SA923, and SA905 with AMPs (AP01518 and AP02380) from APD3 database showed 42.85% and 37.5% similarities, respectively.

From the prediction using the APD3 database, the peptide SA626 shows the closest (at 42.85%) similarity to the EP2 peptide (AP01518) from earthworms. The peptide is determined to be a part of the antibacterial vermipeptide family (AVPF), showing activity against gram-positive and gram-negative bacteria. The SA923 and SA905 show 37.5% similarity to the gageostatin C produced from *Bacillus subtilis,* which has been evaluated to have antimicrobial (against gram-positive and gram-negative bacteria), antifungal, and anticancer activities (Figure 3).

Gageostatin C has been experimentally proven to have antimicrobial activity against important pathogens, such as *Staphylococcus aureus*, *Pseudomonas aeruginosa,* and other plant–fungal pathogens. Furthermore, its SVM score revealed the peptides have the efficiency to target cancer cells. The SA923 peptide has also exhibited a cytotoxic role against different in vitro cancer cell lines [24]. Hence, comparative in silico analysis reveals the potency of the peptide and assists with the development of new strategies to improve its efficacy [25]. Similarly, from all the properties analysed, except the SVM score, the SA923 peptide is predicted, with the highest scores, to be a potential anticancer peptide.

### 3.3. Peptide Family Prediction

The three manually annotated sequences were determined as short peptides with a length of seven amino acids. From the similarity search analysis, SA626 shows similarity to an ABC transporter G family member, early nodulin, and the blue copper protein of *Medicago sativa* with > 85% query coverage and > 70% identity. The proteins are mainly required for the functioning of plants, such as transport of substances, photosynthesis, and nitrogen fixation. Interestingly, SA923 and SA905 show sequence similarity with >50% query coverage and > 100% identity to the pectinesterase inhibitor, chalcone flavone isomerase, vacuolar-processing enzyme, disease-resistance-response protein, and eukaryotic translation initiation factor from different species of the Fabaceae family. The cationic polypeptide from the pectinesterase inhibitor of jelly fig was previously reported for its antitumor activity against human leukemic U937 cells [26]. However, chalcone-flavone isomerase is an important enzyme for biosynthesis of plant flavonoids, with a wide variety of pharmacological applications [27]. Additionally, the peptides have also shown similarity with vacuolar-processing enzymes, where plant protease is apparently known to play a crucial role in various types of cell death in plants [28]. The disease-resistance-response protein of plants offers a platform for interaction of different organisms. Likewise, the plant disease-resistance-response protein and eukaryotic translation initiation factor are significant for protein activation. Thus, from the computational analysis, it is demonstrated that SA923 and SA905 could be the AMPs of plants. However, the SA905 peptide with Pro residue in the seventh position instead of Asp has yielded to similar proteins, as mentioned for the SA923 peptide sequence.

### 3.4. Homology Modelling of the Peptide

**Figure 4:**
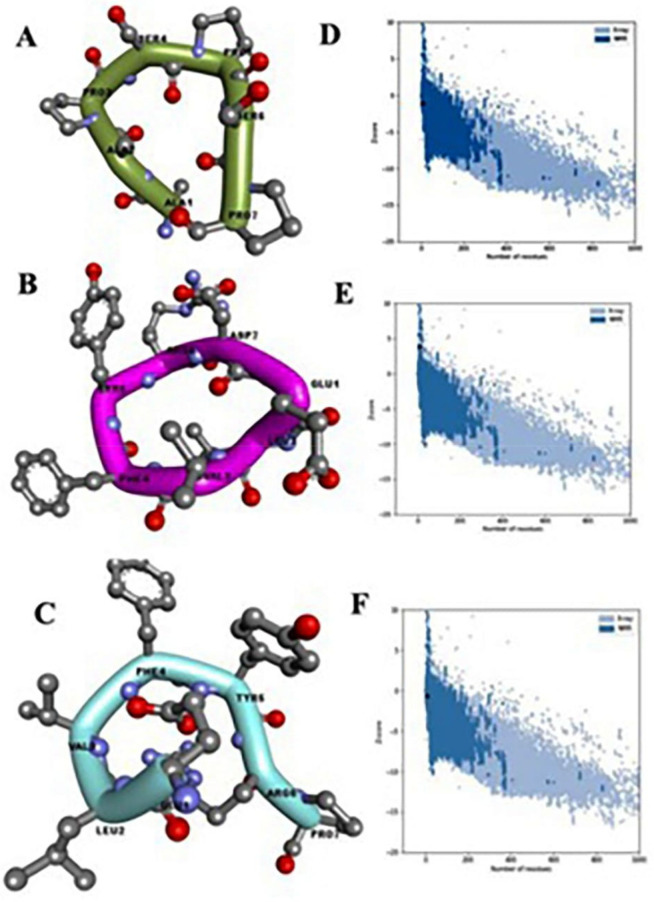
Predicted peptide 3D-structure homology models. (**A**) SA626, (**B**), SA923, and (**C**) SA905 and their corresponding ProSA z^_^score plots (**D**–**F**).

Three-dimensional structures of the short peptides were predicted using PEPstrMOD. The modelled structure was validated using ProSA, and the z-score was determined. From the 3D model, it was observed that SA626 and SA905 showed hairpin-like structure. However, SA923 is the C-N cyclic peptide. The z-scores of SA626, SA905, and SA923 were −1.12, 3.88, and −0.62, respectively. The z-scores of the peptides were in the same ranges as the z-scores of experimentally validated proteins, thus considered to be accurate (Figure 4).

Further structural characterization of the peptide revealed that SA923 is a cyclic peptide with C-N terminal interaction of the carbon atom of ASP7 and nitrogen atom of GLU1 (^1^ELVFYRD^7^). Additionally, SA626 (^1^AAPSPSP^7^) and SA905 (^1^ELVFYRP^7^) have proline, a turn-forming amino acid at the end of their sequences, which might have hindered the cyclization of the sequences. In general, natural cyclic peptides (CPs) are often cyclized by a disulfide bridge (formed by hormones somatostatin, oxytocin, and vasopressin) or by peptide bond (bacitracin). In contrast, SA923 is similar to the family of orbitides, which lack disulfide bonds and instead have small head-to-tail cyclic peptides with proteinogenic amino acids [29]. These CP models are involved in several therapeutic applications, such as antibacterial, antifungal, anticancer, and other properties [30]. Cyclization of peptides can lead to the stiffening of the structure, which has crucial influence on the steric arrangement of the side chains. CPs are predicted to be more stable during physiological conditions than linear peptides and, in contrast, exhibit higher binding potential to the receptor/target proteins [31,32]. Hence, the SA923 CP is expected to have significant biological activity compared to the other peptides.

### 3.5. In Vitro Cytotoxicity Assay

**Figure 5:**
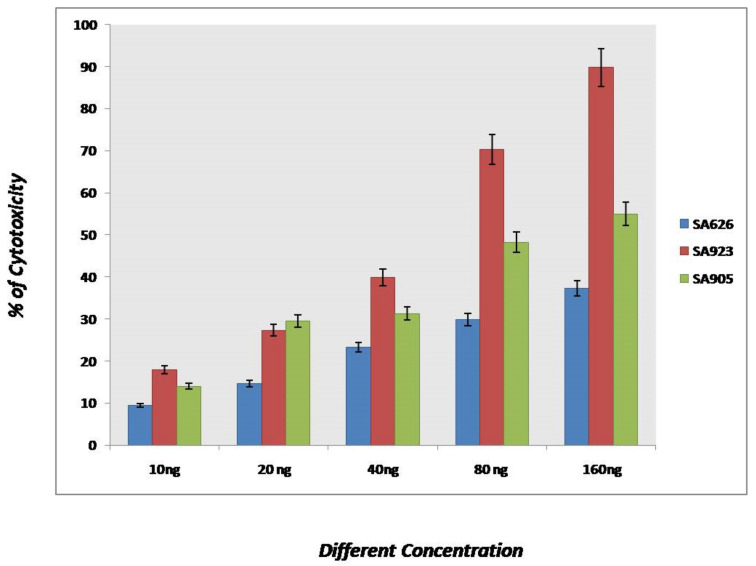
MTT assay for cytotoxicity (%) of peptide in noncancerous 3T3 cells.

An MTT assay was performed to study the cytotoxicity of peptides at different concentrations on 3T3 cells. The peptides (SA626, SA923, and SA905) showed a dose-dependent reduction of cell proliferation (Figure 5). Of all the concentrations, the highest dose of 160 ng/mL showed a promising cytotoxic effect in all the peptide samples.

The SA923 peptide exhibited a potent cytotoxic effect of 89%; when compared to the same dose of SA626, 37%; and SA905, 54%. Thus, the peptides investigated for their in vitro cytotoxicity effect revealed that SA923 has intrinsic ability to inhibit cell proliferation.

### 3.6. Zebrafish Embryo Toxicity Test

**Figure 6:**
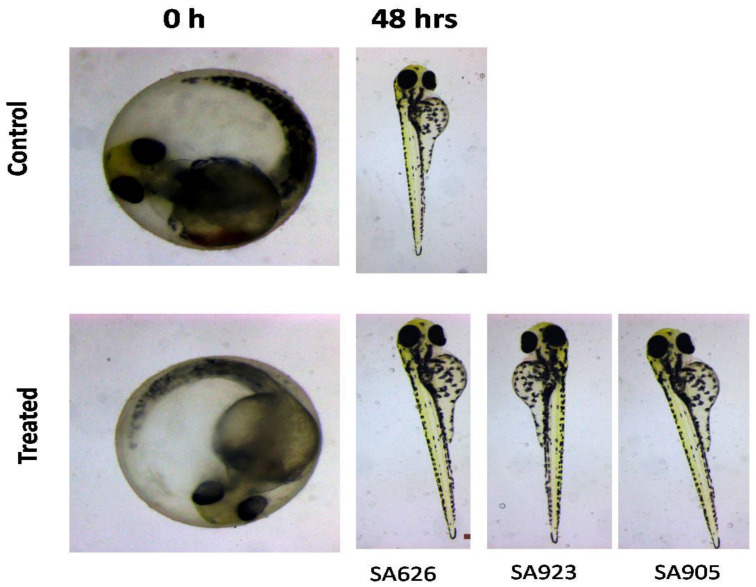
Effects of peptides on zebrafish embryos.

To study the toxicity, zebrafish embryos were subjected to different concentrations of peptides SA626, SA923, and SA905 for 72 h. The embryos were checked at regular intervals for any deformities and death. Embryos exposed to different concentrations did not display any visible anomalies. At 48 h, the embryos in both the control and treated groups reached the larval stage (Figure 6).

**Figure 7:**
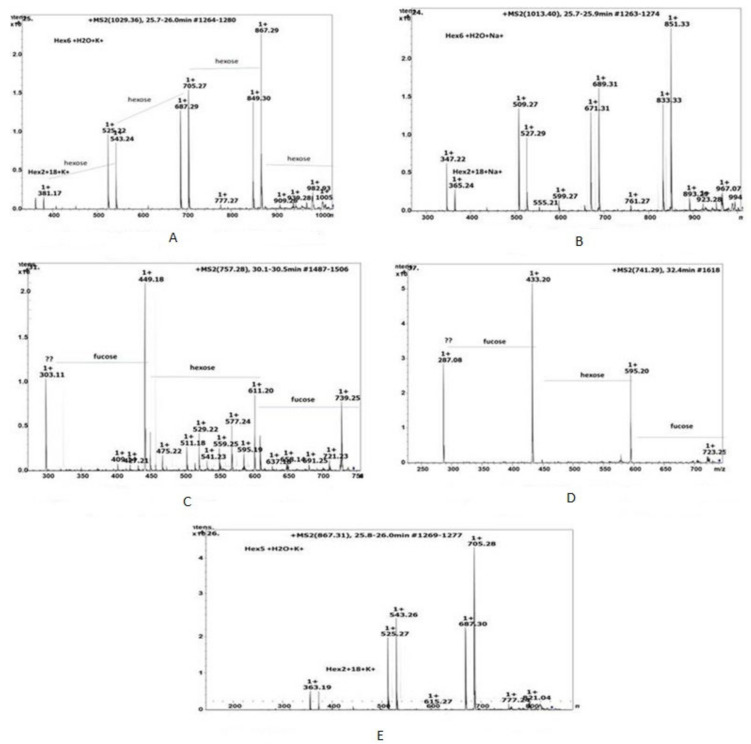
Mass Spectrum of Sugar-based molecules (**A**) SA1029.3 & (**B**) SA 1013.4 with hexose, (**C**) SA 757.2 & (**D**) SA 741.2 with fructose and hexose and (**E**) SA 887.31 with hexose.

Embryonic development is the most important stage for organogenesis in zebrafish. Thus, from the present study, it is determined that peptides from *S. amaranthoides* do not demonstrate any toxic effect on embryos. Peptides promote postfertilization, organogenesis, and hatching of the larvae without any structural abnormalities. The mass spectrum also revealed different sugar-based molecules (SA1029.3, SA1013.4, SA887, SA757.2, and SA741.2), such as multiple hexose and fucose molecules (Figure 7).

Thus, the peptides investigated in this study, having anti-proliferative effects and being non-toxic, are rendered suitable for therapeutic applications. The dose determined in this study is safe and will be a rational amount for the drug-discovery pipeline.

## 4. Conclusions

A total of 86 novel peptides were identified from the natural herb *S. amaranthoides*. Among them, three peptides were characterized, and amino acid sequences were determined using the manual de novo strategy. Based on the computational analysis, SA923 is predicted to be classified as a member of orbitides, which are cyclic peptides. Its physicochemical properties reveal the peptide is stable and has higher binding potential. Additionally, the peptide is predicted to be an anticancer peptide, which was substantiated using an anti-proliferative assay. Hence, detailed investigation of this cyclic peptide may provide insightful direction in pharmaceutical applications.

## Figures and Tables

**Table 1:** Mass-spectrometric identification of peptides from *S. amaranthoides*.

S. No.	Compound	Mz 1^H^	Mz 2^H^
1	Compound **1**	402.54	
2	Compound **2**	436.53	
3	Compound **3**	241.97	
4	Compound **4**	838.26	
5	Compound **5**	920.30	
6	Compound **6**		758.28
7	Compound **7**	851.33	
8	Compound **8**	1013.40	
9	Compound **9**	867.31	
10	Compound **10**	365.25	
11	Compound **11**	257.12	
12	Compound **12**	626.35	
13	Compound **13**	757.28	
14	Compound **14**	595.23	
15	Compound **15**	523.21	
16	Compound **16**	432.40	
17	Compound **17**	476.44	
18	Compound **18**	741.29	
19	Compound **19**	559.22	
20	Compound **20**	355.17	
21	Compound **21**	1087.27	
22	Compound **22**	1117.26	
23	Compound **23**	845.13	
24	Compound **24**	517.16	
25	Compound **25**	499.22	
26	Compound **26**	837.31	
27	Compound **27**	889.51	
28	Compound **28**	923.48	
29	Compound **29**	316.33	
30	Compound **30**	347.24	
31	Compound **31**	411.25	
32	Compound **32**	905.47	
33	Compound **33**	274.28	
34	Compound **34**	675.71	
35	Compound **35**	331.23	
36	Compound **36**	361.16	
37	Compound **37**	661.13	
38	Compound **38**	691.20	
39	Compound **39**	719.24	
40	Compound **40**	429.31	
41	Compound **41**	705.23	
42	Compound **42**	375.22	
43	Compound **43**	443.28	
44	Compound **44**	468.61	
45	Compound **45**	915.36	
46	Compound **46**	483.34	
47	Compound **47**	457.32	
48	Compound **48**	943.43	
49	Compound **49**	957.37	
50	Compound **50**	471.33	
51	Compound **51**	506.34	
52	Compound **52**	279.28	
53	Compound **53**	971.42	
54	Compound **54**	985.39	
55	Compound **55**	456.55	
56	Compound **56**	389.25	
57	Compound **57**	527.26	
58	Compound **58**	999.31	
59	Compound **59**	534.32	
60	Compound **60**	541.27	
61	Compound **61**	717.26	
62	Compound **62**	1027.35	
63	Compound **63**	548.31	
64	Compound **64**	359.25	
65	Compound **65**	555.29	
66	Compound **66**	456.51	
67	Compound **67**	499.33	
68	Compound **68**	574.35	
69	Compound **69**	442.52	
70	Compound **70**	513.42	
71	Compound **71**	597.30	
72	Compound **72**	581.35	
73	Compound **73**	629.49	
74	Compound **74**	297.34	
75	Compound **75**	507.43	
76	Compound **76**	530.49	
77	Compound **77**	283.35	
78	Compound **78**	495.55	
79	Compound **79**	639.33	
80	Compound **80**	613.15	
81	Compound **81**	500.58	
82	Compound **82**	456.62	
83	Compound **83**	607.33	
84	Compound **84**	477.48	
85	Compound **85**	625.37	
86	Compound **86**	609.34	

**Table 2:** List of peptides with their sequences derived using de novo sequencing strategy.

*Peptide*	*Peptide Sequence*	*Residues*	*Mass*
sa626	AAPSPSP	*7*	625.35
SA923	ELVFYRD	*7*	905.47
SA905	ELVFYRP	*7*	923.48

**Table 3:** Physicochemical properties of peptides sourced through computational analysis.

Peptide	Hydrophobic Ratio	Theoretical pI	Stability Index	Aliphatic Index	GRAVY	SVM Score (Anti-cp)	Boman Index (kcal/mol)	z-Score
SA626	28%	5.57	216.66	28.57	−0.400	0.80	0.45	−1.12
SA923	42%	4.37	30.99	97.14	−0.286	0.64	2.66	3.88
SA905	42%	6.10	58.50	97.14	−0.014	0.61	1.41	−0.62

## Data Availability

All the data is provided within the manuscript. The datasets used/or analysed during the current study are available from the corresponding author on reasonable request.

## References

[1] Dashora N., Sodde V., Bhagat J., SPrabhu K., Lobo R. (2011). Antitumor activity of *Dendrophthoe falcata* against ehrlich ascites carcinoma in swiss albino mice. Pharm. Crops.

[2] Ali S.M., Siddiqui R., Khan N.A. (2018). Antimicrobial Discovery from Natural and Unusual Sources. J. Pharm. Pharmacol..

[3] Barashkova A.S., Rogozhin E.A. (2020). Isolation of Antimicrobial Peptides from Different Plant Sources: Does a General Extraction Method Exist?. Plant Methods.

[4] Campos M.L., De Souza C.M., De Oliveira K.B.S., Dias S.C., Franco O.L. (2018). The Role of Antimicrobial Peptides in Plant Immunity. J. Exp. Bot..

[5] Isah T. (2019). Stress and Defense Responses in Plant Secondary Metabolites Production. Biol. Res..

[6] Tang S.-S., Prodhan Z.H., Biswas S.K., Le C.-F., Sekaran S.D. (2018). Antimicrobial Peptides from Different Plant Sources: Isolation, Characterisation, and Purification. Phytochemistry.

[7] Lau J.L., Dunn M.K. (2018). Therapeutic Peptides: Historical Perspectives, Current Development Trends, and Future Directions. Bioorg. Med. Chem..

[8] Fosgerau K., Hoffmann T. (2015). Peptide Therapeutics: Current Status and Future Directions. Drug Discov. Today.

[9] Holaskova E., Galuszka P., Frebort I., Oz M.T. (2015). Antimicrobial Peptide Production and Plant-Based Expression Systems for Medical and Agricultural Biotechnology. Biotechnol. Adv..

[10] Galani V., Patel B., Rana D. (2010). *Sphaeranthus indicus* Linn.: A Phytopharmacological Review. Int. J. Ayurveda Res..

[11] Gayatri S., Maheswara Reddy C.U., Chitra K., Parthasarathy V. (2015). Assessment of in Vitro Cytotoxicity and in Vivo Antitumor Activity of *Sphaeranthus amaranthoides* burm.f. Pharmacogn. Res..

[12] De S., Dey A., Sudhakar Babu A.M.S., Aneela S. (2013). Phytochemical and GC–MS Analysis of Bioactive Compounds of *Sphaeranthus amaranthoides* Burm. Pharmacogn. J..

[13] Reegan A.D., Gandhi M.R., Paulraj M.G., Ignacimuthu S. (2015). Ovicidal and Oviposition Deterrent Activities of Medicinal Plant Extracts against *Aedes aegypti* L. and *Culex quinquefasciatus* Say Mosquitoes (Diptera: Culicidae). Osong Public Health Res. Perspect..

[14] Gayatri S., Suresh R., Reddy C., Chitra K. (2016). Isolation and Characterization of Chemopreventive Agent from *Sphaeranthus amaranthoides* Burm F. Pharmacogn. Res..

[15] Jain R.P., Jayaseelan B.F., Wilson Alphonse C.R., Mahmoud A.H., Mohammed O.B., Ahmed Almunqedhi B.M., Rajaian Pushpabai R. (2021). Mass Spectrometric Identification and Denovo Sequencing of Novel Conotoxins from Vermivorous Cone Snail (*Conus inscriptus*), and Preliminary Screening of Its Venom for Biological Activities in Vitro and in Vivo. Saudi J. Biol. Sci..

[16] Galzitskaya O.V., Kurpe S.R., Panfilov A.V., Glyakina A.V., Grishin S.Y., Kochetov A.P., Deryusheva E.I., Machulin A.V., Kravchenko S.V., Domnin P.A. (2022). Amyloidogenic Peptides: New Class of Antimicrobial Peptides with the Novel Mechanism of Activity. Int. J. Mol. Sci..

[17] Barman A., Deb B., Chakraborty S. (2020). Prediction of Potential Epitopes for Peptide Vaccine Formulation against *Teschovirus A* Using Immunoinformatics. Int. J. Pept. Res. Ther..

[18] Tyagi A., Kapoor P., Kumar R., Chaudhary K., Gautam A., Raghava G.P.S. (2013). In Silico Models for Designing and Discovering Novel Anticancer Peptides. Sci. Rep..

[19] Singh S., Singh H., Tuknait A., Chaudhary K., Singh B., Kumaran S., Raghava G.P.S. (2015). PEPstrMOD: Structure Prediction of Peptides Containing Natural, Non-Natural and Modified Residues. Biol. Direct.

[20] Beema Shafreen R., Seema S., Martinez-Ayala A.L., Lozano-Grande M.A., Robles-Sánchez M., Szterk A., Grishko M., Hanuka E., Katrich E., Gorinstein S. (2019). Binding and Potential Antibiofilm Activities of Amaranthus Proteins against *Candida albicans*. Colloids Surfaces B Biointerfaces.

[21] Geethalakshmi R., Sakravarthi C., Kritika T., Arul Kirubakaran M., Sarada D.V.L. (2013). Evaluation of Antioxidant and Wound Healing Potentials of *Sphaeranthus amaranthoides* Burm.F. Biomed Res. Int..

[22] Rajamohamed B.S., Siddharthan S. (2019). Modulatory Effects of Amukkara Choornam on *Candida albicans* Biofilm: In Vitro and in Vivo Study. Mol. Biol. Rep..

[23] Thanigaivel A., Chanthini K.M.-P., Karthi S., Vasantha-Srinivasan P., Ponsankar A., Sivanesh H., Stanley-Raja V., Shyam-Sundar N., Narayanan K.R., Senthil-Nathan S. (2019). Toxic Effect of Essential Oil and Its Compounds Isolated from *Sphaeranthus amaranthoides* Burm.f. against Dengue Mosquito Vector *Aedes aegypti* Linn. Pestic. Biochem. Physiol..

[24] Gabernet G., Gautschi D., Müller A.T., Neuhaus C.S., Armbrecht L., Dittrich P.S., Hiss J.A., Schneider G. (2019). In Silico Design and Optimization of Selective Membranolytic Anticancer Peptides. Sci. Rep..

[25] Seema S. (2020). Investigation of Potential Antibiofilm Properties of Antimicrobial Peptide (AMP) from Linckia Laevigata against *Candida albicans*: An in Vitro and in Vivo Study. Process Biochem..

[26] Tareq F.S., Lee M.A., Lee H.-S., Lee J.-S., Lee Y.-J., Shin H.J. (2014). Gageostatins A-C, Antimicrobial Linear Lipopeptides from a Marine Bacillus Subtilis. Mar. Drugs.

[27] Okella H., Georrge J.J., Ochwo S., Ndekezi C., Koffi K.T., Aber J., Ajayi C.O., Fofana F.G., Ikiriza H., Mtewa A.G. (2020). New Putative Antimicrobial Candidates: In Silico Design of Fish-Derived Antibacterial Peptide-Motifs. Front. Bioeng. Biotechnol..

[28] Jiang C.-M., Li C.-P., Chang J.-C., Chang H.-M. (2002). Characterization of Pectinesterase Inhibitor in Jelly Fig (*Ficus awkeotsang* Makino) Achenes. J. Agric. Food Chem..

[29] Zhu J., Zhao W., Li R., Guo D., Li H., Wang Y., Mei W., Peng S. (2021). Identification and Characterization of Chalcone Isomerase Genes Involved in Flavonoid Production in *Dracaena cambodiana*. Front. Plant Sci..

[30] Zhang H., Zheng X., Zhang Z. (2010). The Role of Vacuolar Processing Enzymes in Plant Immunity. Plant Signal. Behav..

[31] Fisher M.F., Payne C.D., Chetty T., Crayn D., Berkowitz O., Whelan J., Johan Rosengren K., Mylne J.S. (2020). The Genetic Origin of Evolidine, the First Cyclopeptide Discovered in Plants, and Related Orbitides. J. Biol. Chem..

[32] Gründemann C., Koehbach J., Huber R., Gruber C.W. (2012). Do Plant Cyclotides Have Potential as Immunosuppressant Peptides?. J. Nat. Prod..

